# *Drosophila* to Explore Nucleolar Stress

**DOI:** 10.3390/ijms22136759

**Published:** 2021-06-23

**Authors:** Kathryn R. DeLeo, Sonu S. Baral, Alex Houser, Allison James, Phelan Sewell, Shova Pandey, Patrick J. DiMario

**Affiliations:** Department of Biological Sciences, Louisiana State University, Baton Rouge, LA 70803, USA; kdeleo1@lsu.edu (K.R.D.); sonubaral19@gmail.com (S.S.B.); ahous1@lsuhsc.edu (A.H.); allison.james730@gmail.com (A.J.); psewel3@lsu.edu (P.S.); spand12@lsu.edu (S.P.)

**Keywords:** nucleolar stress, ribosomopathy, *Drosophila*, Nopp140, P bodies, neuroblasts

## Abstract

Nucleolar stress occurs when ribosome production or function declines. Nucleolar stress in stem cells or progenitor cells often leads to disease states called ribosomopathies. *Drosophila* offers a robust system to explore how nucleolar stress causes cell cycle arrest, apoptosis, or autophagy depending on the cell type. We provide an overview of nucleolar stress in *Drosophila* by depleting nucleolar phosphoprotein of 140 kDa (Nopp140), a ribosome biogenesis factor (RBF) in nucleoli and Cajal bodies (CBs). The depletion of Nopp140 in eye imaginal disc cells generates eye deformities reminiscent of craniofacial deformities associated with the Treacher Collins syndrome (TCS), a human ribosomopathy. We show the activation of c-Jun N-terminal Kinase (JNK) in *Drosophila* larvae homozygous for a *Nopp140* gene deletion. JNK is known to induce the expression of the pro-apoptotic Hid protein and autophagy factors Atg1, Atg18.1, and Atg8a; thus, JNK is a central regulator in *Drosophila* nucleolar stress. Ribosome abundance declines upon Nopp140 loss, but unusual cytoplasmic granules accumulate that resemble Processing (P) bodies based on marker proteins, Decapping Protein 1 (DCP1) and Maternal expression at 31B (Me31B). Wild type brain neuroblasts (NBs) express copious amounts of endogenous coilin, but coilin levels decline upon nucleolar stress in most NB types relative to the Mushroom body (MB) NBs. MB NBs exhibit resilience against nucleolar stress as they maintain normal coilin, Deadpan, and EdU labeling levels.

## 1. Introduction

Nucleolar stress occurs when ribosome biogenesis fails [1,2]. Disruptions in ribosome biogenesis can occur at many levels including impairments in rRNA transcription and processing, mutations in ribosomal proteins (RPs), and overall disruptions in nucleolar integrity due to other forms of cell stress such as heat shock or UV irradiation [3]. The ribosome’s catalytic role in protein synthesis and its high energy expenditure during production closely integrate ribosome biogenesis with the overall homeostasis of these cells [4,5]. Therefore, nucleolar stress is particularly detrimental in cells with high demands for protein synthesis such as stem cells, progenitor cells, and cancer cells [2,6].

Mutations in ribosome biogenesis factors (RBFs) or RPs can cause nucleolar stress in select stem cells or progenitor cells, and this often leads to distinct diseases referred to as ribosomopathies [3,7,8,9,10]. For example, in Diamond–Blackfan anemia, adult bone marrow stem cell proliferation is disrupted due to mutations in the genes encoding RPs (RPS19, RPL5, RPL11, RPL15, and others), while other stem cells in the body remain resilient [11,12,13]. Bone marrow failure in this ribosomopathy causes anemia, cranial defects, and a predisposition to cancer [9,11,14]. Similarly, Schwachman–Diamond syndrome is associated with the dysregulation of RP genes (RPS9, RPS20, RPL15, RPL23, and others) leading to bone marrow failure, pancreatic insufficiency, skeletal abnormalities, failures in hematopoiesis, and a greater risk of leukemia [9,15,16]. In yet another example, Treacher Collins syndrome (TCS) results from a loss of defined embryonic neural crest cells that lack sufficient ribosomes due to mutations in the RBF treacle [7,17]. These neural crest cells die by p53-induced apoptosis leading to craniofacial defects [17]. In these various ribosomopathies, only select stem cells or progenitor cells are affected while most of the other stem and progenitor cells within the embryo seem to be unaffected. The question remains: why are certain cells sensitive to nucleolar stress while others remain healthy despite the mutation being systemic?

*Drosophila* offers a robust genetic and cytological system with well described embryology, development, and physiology to study nucleolar stress. *Drosophila* has been a long-time tractable system to study nucleolar organizers, rDNA gene repeats, rRNA, and ribosomes [18,19,20,21]. Phenotypes similar to the ribosomopathies have been studied in *Drosophila* through the deletion of the rDNA genes [22,23,24]. For example, the *bobbed* phenotype occurs when >50% of the rDNA genes are deleted from the *Drosophila* genome. The predominant *bobbed* phenotype causes the shortening and narrowing of posterior scutellar bristles on adult flies. Less frequent *bobbed* phenotypes include reduced body size, etched abdominal tergites, and delayed development. Besides the *bobbed* mutations, *Drosophila* has proven to be an excellent metazoan system to characterize *Minute* mutations (defects in genes encoding the RPs) [8,23,25,26]. These *Minute* mutations are considered ribosomopathies [10].

We have used *Drosophila* to study the effects of nucleolar stress by depleting nucleolar proteins Nucleostemin 1 (NS1) [27], Nucleostemin 2 (NS2) [28], or the early RBF, the nucleolar phosphoprotein of 140 kDa (Nopp140) [29,30,31,32]. Nopp140 is structurally and functionally related to treacle [33]. Vertebrates express both Nopp140 and treacle while *Drosophila* does not encode a close treacle homologue. Depleting Nopp140 in *Drosophila,* however, can model the human TCS. For example, we recently showed that with the loss of Nopp140, mushroom body neuroblasts (MB NBs) in the larval brain are more resilient to nucleolar stress compared to Type I and Type II neuroblasts (NBs) [32], providing an avenue to address the differential sensitivities of various stem cells to nucleolar stress. However, while nucleolar stress in mammalian cells leads to p53-dependent cell cycle arrest or apoptosis [17], we showed that depleting Nopp140 in *Drosophila* initiates p53-independent apoptosis in the larval imaginal wing discs leading to structural deformities in the adult wings [30]. In other words, while we use *Drosophila* as a model system, it is important to keep in mind that there may be differences in nucleolar stress, especially regarding p53 involvement. Thus, pursuing all forms of nucleolar stress in metazoans is critical for our complete understanding. In this report, we confirm and extend our work on nucleolar stress using either RNAi depletion of Nopp140 in various *Drosophila* tissues, a systemic *pBac-*mediated *Nopp140* gene deletion, or a systemic CRISPR-mediated *Nopp140* gene disruption.

## 2. Results

### 2.1. Depletion of Nopp140 in Eye Discs

**Figure 1:**
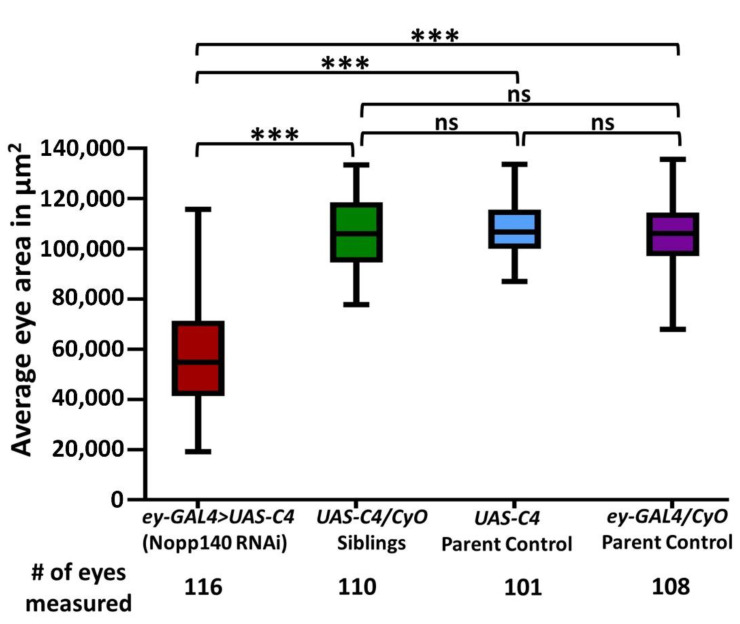
Decrease in eye size due to nucleolar stress generated by the expression of Nopp140-specific RNAi in the *eyeless* (*ey*) pattern. *ey-GAL4/CyO* flies were crossed to homozygous *UAS-C4* flies at 27–28 °C for efficient knockdown of Nopp140. The Nopp140-RNAi expressing flies (*ey-GAL4 > UAS-C4*) with straight wings were compared to parental types and their siblings with curly wings. Data are presented as the average eye area in µm^2^ of all scored flies. The total number of eyes and the number of individual flies scored per genotype are listed at the bottom of the graph. Statistical significance was calculated by an unpaired Student’s *t*-test using GraphPad Prism 9.0.1 with *p* values < 0.0001 indicated as (***).

We showed previously that the depletion of Nopp140 specifically in larval wing discs by RNAi expression (*A9-GAL4 > UAS.C4.2*) resulted in a non-lethal phenotype in which adult wing morphology was perturbed [30]. Pursuing Nopp140 depletion in the eye disc confirms induced morphological defects resulting from Nopp140 depletion in imaginal discs. To examine the effects of Nopp140 depletion in other progenitor diploid cells, especially those of the nervous and visual systems, we used the *eyeless* (*ey*) promoter to drive RNAi expression from RNAi-expressing *UAS-C4* (*ey*-*GAL4 > UAS-C4*) (Figure 1). In the embryo, *ey* is normally expressed in the ventral nerve cord, in primordial cells of the eye imaginal disc [34], and in the embryonic progenitor cells that give rise to the mushroom bodies (MBs) in the central brain lobes [35]. While *ey* is continually expressed in the developing larval eye discs, its expression is restricted mostly to the third larval stage, specifically to the portion of the eye disc that is anterior to the morphogenic furrow [36].

**Figure 2:**
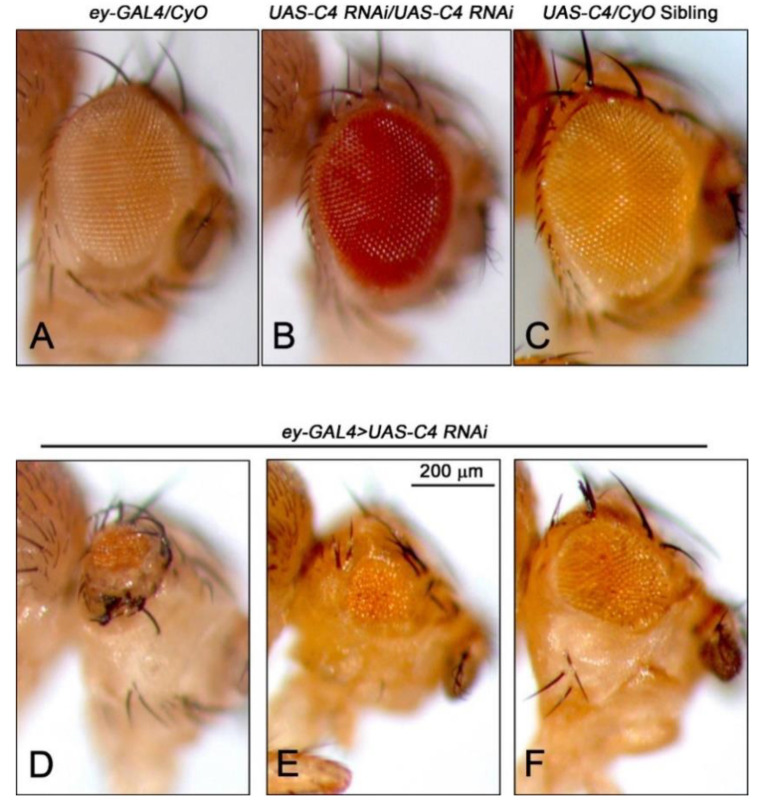
Eye phenotypes caused by nucleolar stress resulting from RNAi depletion of nucleolar phosphoprotein of 140 kDa (Nopp140) in the *ey* expression pattern (*ey-GAL4 > UAS-C4*). Photographs representing eye size and morphology are presented by genotype: parental genotypes were (**A**) *ey-GAL4/CyO* and (**B**) flies homozygous for *UAS-C4,* the transgene encoding shRNA targeting the 5′ end of *Nopp140* transcripts [29]; (**C**) *UAS-C4/CyO* sibling progeny did not express RNAi and, thus, served as controls; and (**D**–**F**) *ey-GAL4/UAS-C4* siblings expressed RNAi. Eye phenotypes in *ey-GAL4/UAS-C4* progeny were variable. Bar = 200 µm for all images.

Parental fly lines were crossed to generate RNAi-expressing *(ey-GAL4 > UAS-C4)* adults with straight wings and *UAS-C4/CyO* control siblings with curled wings. The majority of Nopp140-RNAi expressing flies displayed eyes that were malformed and relatively small (Figure 1 and Figure 2D–F), measuring almost half the area of eyes in parental flies and sibling controls (Figure 1). The eye phenotypes of Nopp140-RNAi expressing flies, however, varied greatly from a mild almost wild type size to a severe reduction in size displaying very few ommatidia. This wide phenotypic distribution is represented in the graphed data points for the *ey-GAL4 > UAS-C4* in Figure 1. In addition, we often observed a change in the pattern of bristles that normally protrude between the ommatidia; these bristles were short and non-uniform. The cuticle hairs that normally surround the eye were also misshapen in most RNAi-expressing flies (Figure 2E,F). One of the more unusual eye phenotypes had ectopic tissues protruding from the eye itself, suggesting that the normal patterning of the eye-antennal discs may have been disrupted during larval development (Figure S1).

### 2.2. Stress-Related Proteins Are Upregulated in Homozygous Nopp140 Gene Knockout Larvae

**Figure 3:**
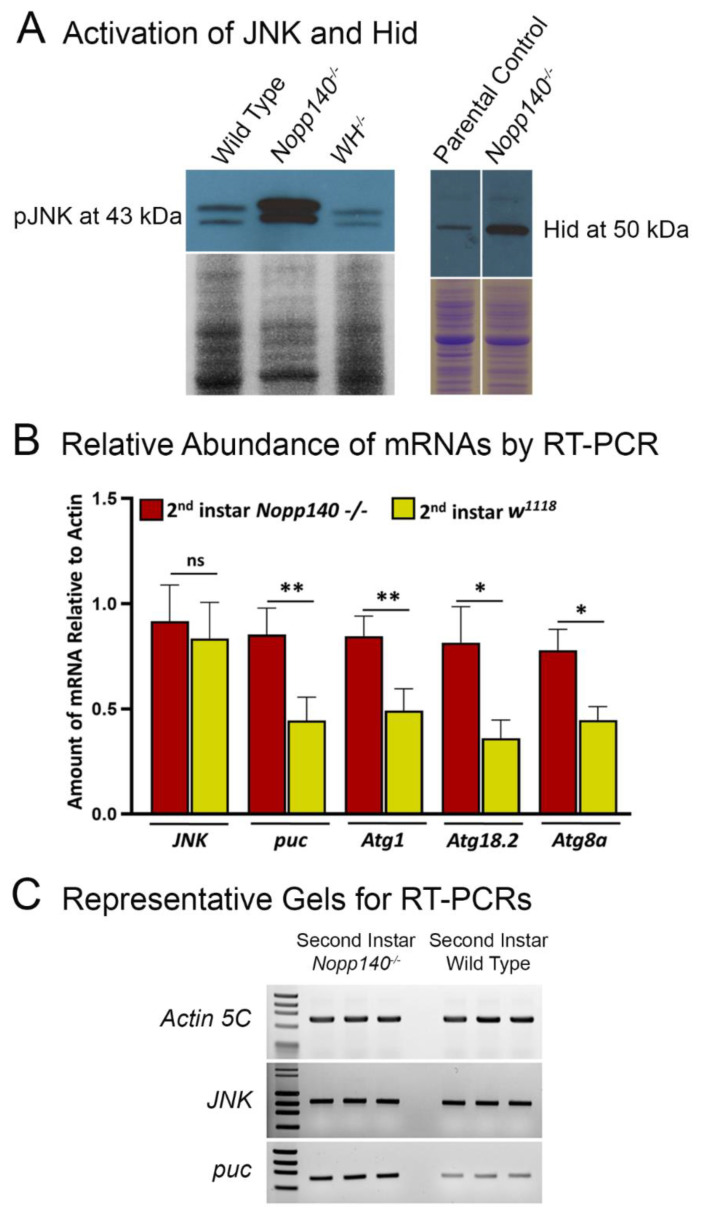
Activation of the JNK pathway in *Nopp140-/-* knockout larvae. (**A**) The left-hand western blot shows activation of c-Jun N-terminal kinase (JNK) as detected by an antibody against phospho-JNK in *KO121 Nopp140-/-* larval extracts versus wild type and WH-/- larval extracts. WH- refers to the original fly line containing the *pBac* element (*f04633*) with the WH- orientation and located in the 3′ coding sequence of *P5CDH1* positioned immediately downstream of *Nopp140* and used to delete the *Nopp140* gene (see [31]). The right-hand western blot shows accumulation of pro-apoptotic Hid compared to parental controls. JNK phosphorylates AP-1 which then activates the *hid* gene. Companion Coomassie stained gels are included below each Western blot as loading controls. (**B**) While JNK kinase increased in *KO121 Nopp140-/-* larvae compared to similarly aged (2nd instar) wild type (*w^1118^*) controls, RT-PCRs showed, as expected, that *JNK* transcript levels were not significantly different between *KO121 Nopp140-/-* and wild type (*w^1118^*). Expression of *puc* is a commonly used indicator of JNK kinase activity. Abundance of autophagy transcripts *Atg1*, *Atg18.2*, and *Atg8a* were also elevated. Data are presented as average RT-PCR products of individual transcript levels compared to that of *Actin5C*. Error bars represent mean ± standard deviation of at least three independent determinations and statistical significance was calculated by a paired Student’s *t*-test using GraphPad Prism 9.0.1 with *p* values < 0.05 are indicated (*) and with *p* values < 0.005 are indicated (**). (**C**) Representative agarose gels show *Actin5C* (as a standard control), *JNK*, and *puc* RT-PCR products. Four independent experiments were performed per gene, and PCR reactions were performed in triplicate.

Previous immunoblot results indicated that c-Jun N-terminal kinase (JNK) was activated and that the pro-apoptotic Hid protein was expressed in the larvae depleted for Nopp140 by RNAi expression [30]. We subsequently deleted the *Nopp140* gene by recombination between flanking RB+ and WH- *pBac* elements. One of the *Nopp140* deletion lines is referred to as *KO121* [31]. We used Western blots and an antibody directed against activated JNK (pJNK) (Cell Signaling) to show that JNK is activated in larvae homozygous for the *KO121 Nopp140* gene deletion (Figure 3A). Wild type larvae (*w^1118^*) and larvae homozygous for the downstream *pBac* element (*WH-/-*) used to delete *Nopp140* served as controls. We often detected two JNK bands at about 43 kDa as shown in Figure 3A but would just as often see a single JNK band (Figure S2) [37,38,39]. As far as we know, *Drosophila* has just one isoform of JNK, so we attribute the two bands in Figure 3A to partial proteolysis. In addition to JNK activation, the pro-apoptotic Hid protein was upregulated in the *KO121 Nopp140-/-* larvae (Figure 3A). We also observed Hid induction in larvae depleted for Nopp140 by RNAi expression (Figure S2), confirming the onset of apoptosis either by RNAi depletion of Nopp140 or by *KO121 Nopp140* gene knockout.

We next performed RT-PCR measurements using specific primers targeting transcripts that encode *JNK*, *puc*, several autophagy markers, and *Actin5C* as a control (Figure 3B,C). The quantitation results are presented as a ratio of gene-specific RT-PCR product normalized to the *Actin5C*-specific product (Figure 3B). *JNK* transcript levels were near equivalent in all samples, as expected for JNK’s expression and regulation indicating that nucleolar stress in *Drosophila* activates the JNK protein by phosphorylation rather than by inducing the *JNK* gene. Activated JNK (pJNK) phosphorylates the transcription factor AP-1. AP-1 then activates the expression of the pro-apoptotic *hid* gene [40,41] and the *puc* gene which encodes a phosphatase that down-regulates JNK’s activity in a negative feedback loop. Compared to similarly aged wild type (*w^1118^*) larvae, *puc* gene expression in *KO121 Nopp140*-/- was significantly increased (Figure 3B,C). Measuring the amount of *puc* mRNA present in *Drosophila* larvae has often been used as an indirect yet common assessment of JNK’s phosphorylation/activity status [41,42].

We previously showed the formation of autophagosomes in larval polyploid gut cells due to nucleolar stress [27,28,30]. Here, we conducted RT-PCRs on whole larval RNA extracts to assay the expression of three individual genes involved in the autophagy pathway [42]. Atg1 is a kinase that forms a complex responsible for the initiation of phagophore formation, and Atg18.2 is responsible for further assembly of the phagophore. Atg8a is a ubiquitin-like protein, conjugated to phosphatidyl-ethanolamine (PE) in both the inner and outer autophagosome membrane leaflets [42,43]. Compared to similarly aged wild type controls (*w^1118^*), the expression of *Atg1*, *Atg18.2*, and *Atg8a* was significantly increased in *KO121 Nopp140-/-* samples (Figure 3B).

### 2.3. Processing(P)-Like Granules Appear in Polyploid Midgut of Nopp140-/- Larvae

**Figure 4:**
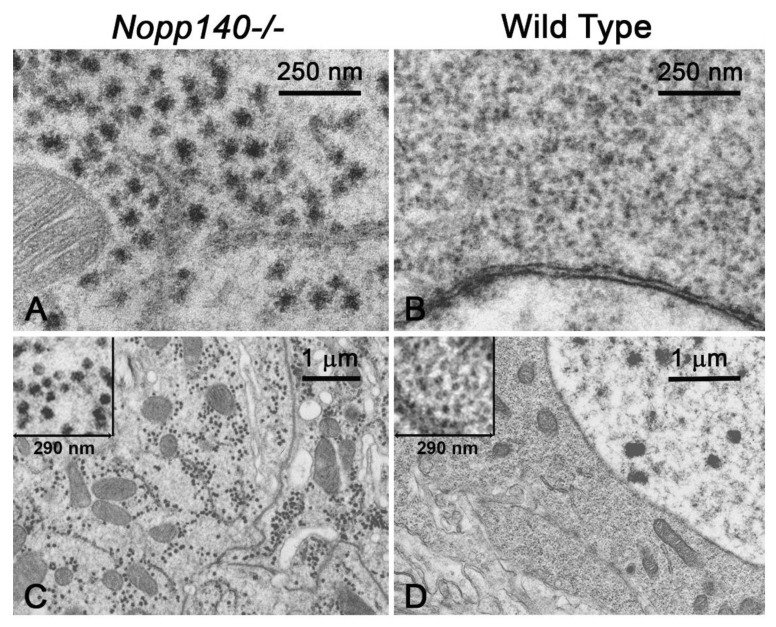
Ultrastructural examination of midgut cells from *KO121 Nopp140-/-* and wild type larvae. (**A**) The midgut cell cytoplasm from a 3–4-day old *KO121 Nopp140-/-* larva lacked ribosomes but contained numerous electron dense granules each consisting of a 35–45 nm diameter core with ribosomes tethered to their periphery. A mitochondrion is on the left. (**B**) For comparison, the cytoplasm of a wild type midgut cell contained copious ribosomes. The nuclear envelope occupies the bottom portion of the image. (**C**) The cytoplasm of a 6-day old *KO121 Nopp140-/-* larval midgut cell showed only the cores with very few ribosomes left in the cell. Many of the core granules appeared in clusters. (**D**) For comparison, a wild type midgut cell. The nucleus occupies the upper right-hand portion of the image. (**C**,**D**) The insets provide high magnification views of 290 nm square areas.

We initially used transmission electron microscopy (TEM) to assess ribosome abundance in larval cells lacking Nopp140 [30,31]. While ribosome abundance appeared diminished in the relatively few cell types we examined, we observed unusual electron dense granules in the cytoplasm of polyploid midgut cells. *KO121 Nopp140-/-* larvae arrest in the second instar larval stage and linger for days. In larvae that were 3–4 days old, the granules appear to have a central core diameter of approximately 35–45 nm but with ribosomes attached to the periphery of the core (Figure 4A). As the *KO121 Nopp140-/-* larvae aged, they were still arrested in the second instar larval stage, but the number of peripheral ribosomes associated with the granules diminished, leaving only the cores (Figure 4C). At this point, there were essentially no ribosomes left in these midgut cells. Many of the granules appeared in clusters, but the granules remained separate without indication of fusing to form larger bodies (inset for Figure 4C). Wild type midgut cells are shown to provide stark contrasts in ribosome abundance (Figure 4B,D). The inset in Figure 4D shows a similar high magnification view of the cytoplasm from a wild type midgut cell. Ribosomes free in the cytoplasm or attached to the endoplasmic reticulum (lower left in the inset) are visible.

By TEM analysis, the abundance of the dense granules is greatest in midgut cells from *Nopp140-/-* larvae homozygous for either the *KO121* allele or the CRISPR-disrupted *J11* allele. We have seen the dense granules in relatively low abundance in neurons of the ventral nerve cord in *KO121 Nopp140-/-* larvae and rarely in the neuronal cells of their central brain lobes (Figure S3A). A partial depletion of Nopp140 by RNAi generated relatively few granules in midgut cells (Figure S3B).

**Figure 5:**
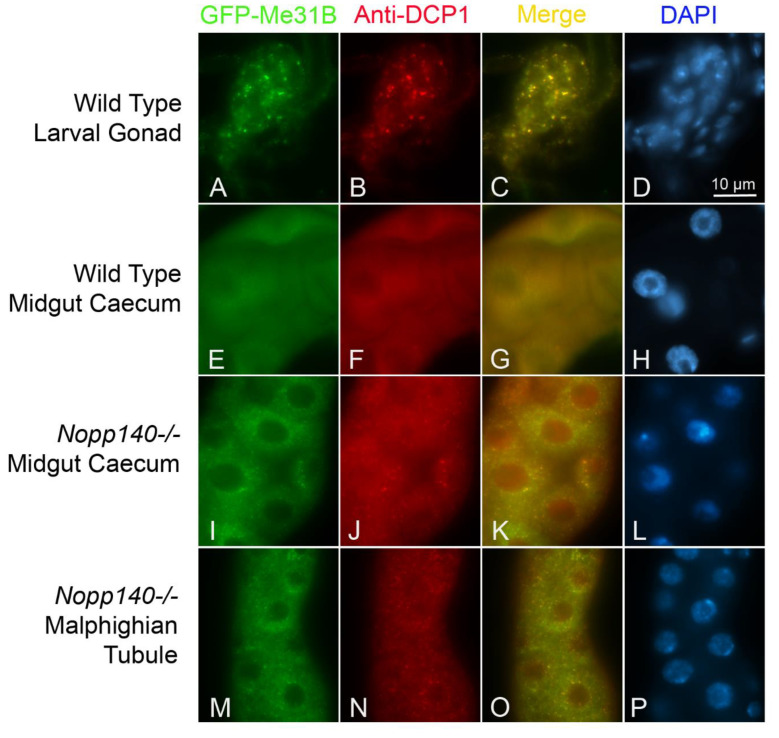
Nucleolar stress-induced midgut granules contain Processing (P) body markers: Maternal expression at 31B (Me31B) and Decapping Protein 1 (DCP1). GFP-Me31B was a GFP protein trap fusion expressed from the endogenous *Me31B* gene promoter. DCP1 was detected by immuno-fluorescence. (**A–D**) As a positive control, GFP-Me31B and DCP1 colocalized in P bodies present in wild type larval gonads. (**E–H**) As a negative control wild type midgut caecum contained the two P body markers, but they were diffusely distributed throughout the cells with no apparent formation of P bodies under non-stress conditions. ((**I–L, M–P**), respectively) The *KO121 Nopp140-/-* midgut caecum and Malpighian tubules displayed colocalization of GFP-Me31B and DCP1 in the cytoplasmic granules supporting their identity as putative P bodies. Bar = 10 µm for all images.

In attempts to identify these granules, we used an antibody directed against *Drosophila* Decapping Protein 1 (DCP1) [44] along with a *Drosophila* protein trap line in which Maternal expression at 31B (Me31B) is tagged with GFP. Both DCP1 and Me31B are markers for Processing (P) bodies [45]. As a positive control, anti-DCP1 and GFP-Me31B colocalized within maternal mRNP granules known to reside normally within the germ cells of larval gonads as seen in the protein trap line (Figure 5A–C) (see [46]). We also note that brain cells of larvae from the protein trap line normally contain numerous mRNP granules labeled by both GFP-Me31B and anti-DCP1 (Figure S4) (see [47]). As a negative control, we examined the distribution of GFP-Me31B and DCP1 in midgut caecum cells of larvae from the protein trap line expressing GFP-Me31B, and as expected, we saw no cytoplasmic granules (Figure 5D–F). However, when genetically combined with the *KO121 Nopp140*-/- gene knockout, GFP-Me31B and anti-DCP1 colocalized to numerous cytoplasmic foci in midgut caecum cells (Figure 5G–I) and Malpighian tubule cells (Figure 5J–L). We note a redistribution of Me31B from the nucleus to the cytoplasm in these stressed cells. The observation suggests that formation of P bodies in the midgut cells as defined by Me31B and DCP1 correlates with the appearance of the electron dense granules at the ultrastructural level.

### 2.4. Coilin Expression in Larval Neuroblasts

**Figure 6:**
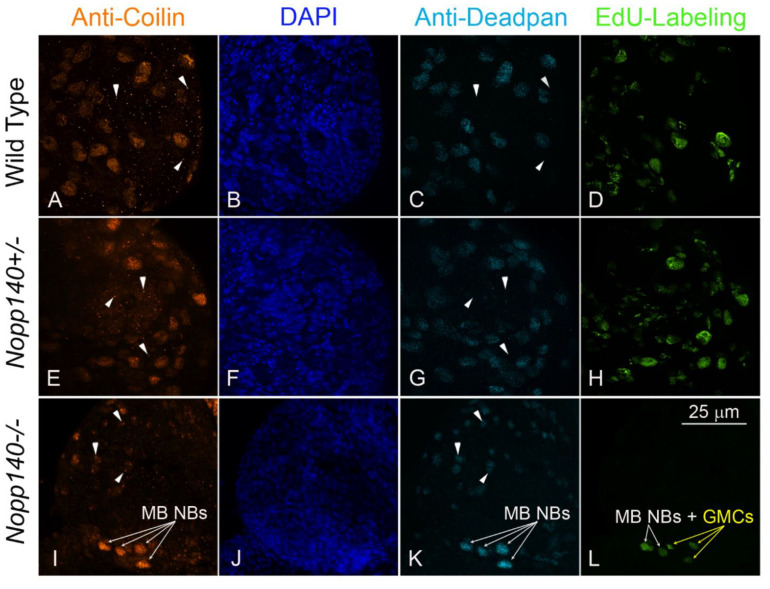
Mushroom body neuroblasts (MB NBs) displayed resilience amid nucleolar stress. In larval brains (**A,E,I**) anti-coilin labeled coilin within nuclei, (**B,F,J**) DAPI labeled nuclear DNA, (**C,G,K**) anti-Deadpan labeled nuclei of only the neuroblasts (NBs), and (**D,H,L**) a half hour pulse of EdU labeled S-phase cells. (**A,E**) In wild type and *Nopp140+/-* larval brains, coilin localized to Cajal bodies (CBs) in neurons (white arrow heads) but throughout the non-nucleolar nucleoplasm in NBs as identified by Deadpan labeling (**C,G**). S-phase NBs and ganglionic mother cells (GMCs) throughout the brain were EdU positive in both wild type (**D**: *w^1118^*, *n* = 15) and *Nopp140+/-* (**H**, *n* = 12) larvae. In the one *J11 Nopp140-/-* larval brain shown here (**L**, *n* = 9), two of the four MB NBs (white arrows) and the MB lineage GMCs (yellow arrows) were labeled with EdU. (**K**) Anti-Deadpan labeled all four MB NBs (white arrows) and confirmed the identity of the EdU positive MB GMCs by their proximity to the MB NBs. (**I**) In the *J11 Nopp140-/-* brain, the MB NBs (white arrows) maintained a wide expanse of coilin throughout the non-nucleolar nucleoplasm, while other NBs had comparatively less nucleoplasmic coilin but showed now discernable CBs (white arrow heads). Bar = 25 µm for all images.

We showed previously that the four MB NBs in each larval brain lobe maintain a marked resilience toward nucleolar stress in *J11 Nopp140-/-* larvae [32]. These MB NBs contain relatively high levels of Deadpan, a transcription factor and marker unique to NBs [48], while most other NBs showed a partial or complete loss of anti-Deadpan labeling (Figure 6, compare panels C, G, and K). We note, however, that anti-Deadpan labeling of other *J11 Nopp140-/-* NBs diminishes over time [32]. The MB NBs and their descendent ganglionic mother cells (GMCs) in *J11 Nopp140-/-* larvae also label well with EdU (Figure 6, compare panels D, H, and L), while most other cells in the *J11 Nopp140-/-* brain fail to do so. This indicates that the MB NBs and their GMCs in *J11 Nopp140-/-* larvae remain in the cell cycle. As expected, wild type and heterozygous *J11 Nopp140+/-* larval brains displayed many more NBs labeled by anti-Deadpan (Figure 6C,G), and far more EdU-positive cells (Figure 6D,H).

Since Nopp140 locates to nucleoli and Cajal bodies (CBs) [49,50], we wanted to determine if CB integrity was affected by nucleolar stress. Coilin is the unique protein marker for CBs [51,52], so using an antibody directed against *Drosophila* coilin [53], we first found endogenous coilin extensively expressed and distributed in the nucleoplasm outside of the nucleolus in both wild type and *Nopp140+/-* NBs as defined by their coincident anti-Deadpan labeling (Figure 6A,C,E,G). We also found distinct CBs in what should be neurons in wild type and *J11 Nopp140+/-* heterozygous larvae (arrow heads in Figure 6A,E). In *J11 Nopp140-/-* larval brains, MB NBs maintained high levels of nucleoplasmic coilin, while coilin abundance was comparatively reduced in the remaining cerebral NBs, again identified by their co-labeling with anti-Deadpan (Figure 6I,K). The majority of these *J11 Nopp140-/-* NBs with the exception of the MB NBs, now showed distinct CBs instead of diffuse coilin distribution throughout the nucleoplasm. This observation supports our hypothesis that the MB NBs are more resilient to nucleolar stress generated by the loss of Nopp140 than other NB types in the larval brain [32].

## 3. Discussion

Previously, we induced nucleolar stress in the larval imaginal wing discs with phenotypic readout in adult wing morphology. This allowed us to show that apoptosis resulting from nucleolar stress in the larval wing disc was p53-independent but associated with JNK activation [30]. Here, we used the UAS-GAL4 system to express RNAi to deplete Nopp140 in the larval eye disc. This restricted depletion of Nopp140 avoided organismal lethality but provided phenotypic read outs that mimic the human ribosomopathy TCS in which the related RBF, treacle, is mutated. The phenotypic variability we saw in eye development upon the depletion of Nopp140 is reminiscent of the phenotypic variability in the TCS [7,9].

In this report, we used our original *KO121 Nopp140* gene deletion [31] to support our claim of enhanced JNK activation upon RNAi depletion of Nopp140. We showed that the expressions of the pro-apoptotic factor Hid (Figure 3A) and puc (Figure 3B) were both enhanced upon JNK activation (Figure 3A,B), as expected [54,55].

We previously described autophagy in midgut and hindgut polyploid cells due to nucleolar stress generated by the partial losses of NS1 and NS2 [27,28] or Nopp140 [30] by RNAi. Here, we showed the enhanced expression of Atg1, Atg18.2, and Atg8a by RT-PCR in larvae homozygous for our original *KO121 Nopp140* gene deletion. An apparent discrepancy exists between nucleolar stress generated by the RNAi depletion of NS1, NS2, and Nopp140 compared to the complete loss of Nopp140 by gene deletion; specifically, we did not see a robust formation of autophagosomes by TEM in cells completely lacking Nopp140 as we saw with the partial losses of NS1/NS2 and Nopp140 by RNAi. One possibility to explain this discrepancy is that cells partially depleted of NS1/NS2 and Nopp140 maintain enough cytoplasmic ribosomes such that autophagy factors can be translated. Conversely, cells completely lacking Nopp140 may have far too few ribosomes (e.g., Figure 4C) resulting in a failure to translate autophagy factors despite the production of their mRNAs (Figure 3B). Further work is necessary to define precisely when and in which cell types autophagy is induced in response to nucleolar stress resulting from the type and extent of RBF loss.

Figure 4 described unusual electron dense bodies that form in the cytoplasm of primarily midgut cells in larvae homozygous for the *Nopp140* deletion allele (*KO121*) or the CRISPR disruption allele (*J11*). The ultrastructure of the granules changed with larval age. The granules in young larvae had an electron dense central core with ribosomes tethered to the periphery (Figure 4A). By day 6, the midgut cells were starkly depleted for ribosomes, and the granules consisted of just the core without attached ribosomes (Figure 4C). We found similar granules in neuronal cells of the ventral nerve cord and central brain lobes of *KO121 Nopp140-/-* cells, but not as frequently on a cell-by-cell basis nor in high numbers on a per cell basis as in the midgut. Whether in young or older larvae, the granules remained separate from each other but were often found in clusters. By fluorescence microscopy (Figure 5), we showed that Nopp140-deficient midgut cells contained P-like bodies with established markers, Me31B and DCP1 [45,46,47,56,57,58]. Naturally occurring RNP granules that contain DCP1 and Me31B in neuronal cells precluded efforts to determine if P bodies formed in these cells due to the loss of Nopp140. We are currently marking DCP1 and Me31B for co-relative light and electron microscopy to test if the electron dense granules seen by TEM are in fact the P bodies detected by fluorescence microscopy. While this has yet to be determined, we hypothesize that P bodies would form to degrade mRNA associated with perhaps defective ribosomes assembled in the absence of Nopp140. At this time, we cannot rule out other possibilities such as a link between the dense granules and proteosomes.

### 3.1. Working Model

**Figure 7:**
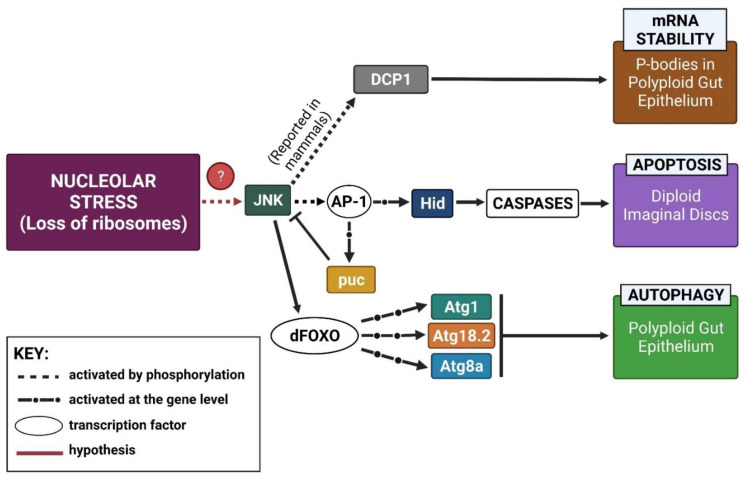
Our current working model of nucleolar stress in *Drosophila* induced by the loss of ribosome biogenesis factors. Various cell stress responses are tissue specific, but all are predicted to be JNK-dependent. Autophagy was visible by TEM in the *Drosophila* gut epithelium upon loss of NS1 and NS2 [27,28] while apoptosis occurred in diploid imaginal wing disc cells upon loss of Nopp140 [30]. This report showed that upon complete loss of Nopp140 by gene deletion, JNK was activated leading to the up-regulations in puc, Hid, Atg1, Atg18.2, and Atg8a. We know that activated JNK regulates the formation of P bodies in mammalian cells by phosphorylating DCP1 [59]. We have yet to establish this JNK-DCP1 link in *Drosophila* and to link the activation of JNK to nucleolar stress via several different upstream kinases.

Our tentative model for the nucleolar stress pathway in *Drosophila* (Figure 7) is based on previous observations [30,31] but supported and expanded by the data presented here. We previously showed apoptosis in imaginal wing discs and autophagy in polyploid gut tissue upon Nopp140 depletion by RNAi [30]. The apoptosis in the wing disc was JNK-dependent, but p53-independent. Apoptosis in a p53-independent manner was previously established in *Drosophila* [41]. Thus, the activation of JNK may be the central link between nucleolar stress and either apoptosis or autophagy, depending on the cell type. The activation of JNK was confirmed by the enhanced expression of puc (Figure 3B,C), a negative regulator of JNK [54]. We also know from the work of Shlevkov and Morata [55] that JNK activates the pro-apoptotic *hid* gene, and similarly, we noted the enhanced expression of Hid in larvae homozygous for *pBac*-generated *KO121 Nopp140-/-* (Figure 3A) and again by RNAi (Figure S2) Interestingly, activated JNK regulates P body formation in mammalian cells by phosphorylating DCP1 [59]. Future work on these nucleolar stress-induced granules will further establish their identity as P bodies and determine the relationship between activated JNK and formation of these granules in *Drosophila*. Fortunately, there is only a single version of JNK in *Drosophila*, unlike mammalian systems which have several. This single JNK should allow us to pursue the equally important question of what upstream effectors (kinases) respond to nucleolar stress in *Drosophila* to activate JNK. We hope to verify the pathway by knocking down effectors upstream and downstream of JNK in various cell types and at different times in development.

### 3.2. Mushroom Body Lineages in Nopp140-/- Larvae Show Resilience to Nucleolar Stress

While coilin typically locates to CBs and is responsible for CB integrity [53,60,61], we found coilin distributed throughout the nucleoplasm outside of the nucleolus in NBs of both wild type and heterozygous *J11 Nopp140+/-* larvae. Liu et al. [53] also demonstrated high nucleoplasmic but non-nucleolar coilin expression without discernable CBs in germline stem cells of both the ovary and testis in *Drosophila*. Thus, we hypothesize that coilin localization changes from CBs to non-nucleolar nucleoplasm in certain stem cell populations due to the excessive expression of endogenous coilin in these cells. CB identification by coilin labeling alone is then no longer possible in these stem cell populations as coilin is no longer exclusive to the CB [60]. In *J11 Nopp140*-/- larvae, all NBs barring the MB NBs lack diffuse coilin distribution perhaps due to reduced coilin expression that results from a loss of ribosomes and reduced overall protein synthesis. Residual coilin now locates to only the CBs in these NBs.

We showed earlier [32] that the four MB NBs per larval brain lobe in *J11 Nopp140-/-* display a resilience to nucleolar stress caused by the depletion of Nopp140. We are currently investigating a few hypotheses that could explain this resilience. First, we predict that the ribosome content in these MB NBs is high compared to other NBs in *J11 Nopp140-/-* larval brains. If so, how would these levels be maintained? One possibility is that maternally encoded RBFs perdure longer in the MB lineages than in other NBs lineages. While other NB lineages enter a normal programed quiescence at the embryo-larval transition, the MB NBs maintain mitotic and metabolic activity during this time likely generating a continuous supply of ribosomes. We want to test by clonal analysis if these MB NBs stockpile RBFs by translation of remaining maternal transcripts during this period of quiescence for the other NBs. These accumulated RBFs (e.g., Nopp140, fibrillarin) in the MB NBs would allow the continued production of zygotic ribosomes further into the larval stages. While we cannot dismiss the possibility that the MB NBs maintain maternal ribosomes versus producing new zygotic ribosomes, we see this possibility as less likely because we expect equivalent lifespans of maternal ribosomes in the various NBs. Finally, maintaining coilin abundance in the MB NBs upon the loss of Nopp140 suggests that the entire nucleolus-CB axis of ribosome production is maintained in these cells as compared to the other NB varieties. Our future goal is to define the molecular basis for differential sensitivities or resilience to nucleolar stress in the various *Drosophila* NB lineages. This understanding should better explain why ribosomopathies affect some stem cells and progenitor cells but not others and provide further insight into using nucleolar stress as a treatment for cancer.

## 4. Materials and Methods

### 4.1. Fly Stocks

All *Drosophila melanogaster* stocks were grown on standard cornmeal and molasses fly food at room temperature. GAL4 drivers included P{w^+m *^ = GAL4-ey.H}4-8/CyO (Bloomington stock 5535), abbreviated here as *ey-GAL4*/*CyO* and *P{w^+mW.hs^] = GawB}OK107 ey^OK107^/In(4)ci^D^, ci^D^ pan^ciD^ sv^spa-pol^* (Bloomington stock 854), abbreviated here as *OK107-GAL4*). The original P{w^+mC^ = *UAS-Nopp140.dsRNA}^C4^* RNAi line described by [29] was used to deplete Nopp140. It contains two RNAi-expressing transgenes, one on the second chromosome and the other on the third chromosome. This stock, referred to as *UAS-C4*, is homozygous viable and fertile. The *GFP-Me31B* protein trap line was *P{w^+mC^ = PTT-GB}Me31B^CB05282^* (Bloomington stock 51530). The *pBac*-mediated *Nopp140* gene deletion line, *KO121*, was described by He*,* et al. [31]. The CRISPR-mediated *Nopp140* gene disruption line, *J11,* was described by Baral, Lieux and DiMario [32]. The *w^1118^* line served as a wild type control (Bloomington stock 3605).

### 4.2. Assessing Eye Morphology

Homozygous *UAS-C4* flies were crossed to *ey-GAL4/CyO* flies. F1 progeny were then selected based on their wing phenotype; straight winged flies expressed RNAi to deplete Nopp140, while curly-winged siblings served as a control. Flies were imaged using a Lumar.V12 SteREO microscope (Zeiss, Jena, Germany) fitted with an Axiocam MRc5 camera (Zeiss, Jena, Germany). To measure eye size, a line was drawn around visible ommatidia using the contour (spline) function on the ZEN 2 Pro Software (Zeiss). The data are presented as average eye size (measured in μm^2^) per genotype.

### 4.3. RT-PCRs

Total RNA was extracted from whole larvae with TRIzol^®^ as recommended by the manufacturer. All samples were treated with DNase I (NEB). RNA concentration was measured by Nanodrop (ThermoScientific, Waltham, MA, USA), and 2 µg of total RNA was used in each RT reaction using M-MLV reverse transcriptase (Promega) with an oligo-dT to amplify all poly(A+) mRNAs present in sample. To ensure against any genomic DNA contamination, duplicate RT-PCR experiments were performed without the RT enzyme. After the RT reaction, all samples were treated with RNase H (NEB) and RNase cocktail (Ambion, Cat. #AM2286). One µL of the RNA-free RT reaction was used as template per PCR reaction. PCR reactions were carried out using *Taq* Polymerase (NEB) with ThermoPol Buffer. The number of amplification cycles necessary to produce a measurable product varied between genes: *Actin5C* (25 to 30 cycles), *JNK* (26 to 30 cycles), *puc* (26 cycles), A*tg1,* and *Atg18.2* (27 cycles), and *Atg8a* (22 to 24 cycles). *Atg1, Atg18.2*, and *Atg8a* primer sequences were from Wu, Wang and Bohmann [42]. The forward (F) and reverse (R) primer sets were as follows: *Atg1* (F) 5′-GAGTATTGCAATGGCGGCGACT-3′ and (R) 5′-CAGGAATCGCGCAAACCCAA-3′, with an expected product of 243 nt.; *Atg18.2* (F) 5′-CCGAAAATTCTGCCAAGGAAGC-3′ and (R) 5′-AATCCGTCCTCGCACGCAAT-3′, with an expected product of 250 nt.; *Atg8a* (F) 5′-GCAAATATCCAGACCGTGTGCC-3′ and (R) 5′-AGCCCATGGTAGCCGATGTT-3′, with an expected product of 210 nt.; *JNK* (F) 5′-CACCATGACGACAGCTCAGCACC-3′ and (R) 5′-CTACCGCGTTCTATTATTTGTATTGTGTGCTTCG-3′, with an expected product of 1,123 nt.; *puc* (F) 5′ GATTTCGCGGAGCGCCACCATTGC 3′ and (R) 5′ TCAGTCCCTCGTCAAATTGCTAGCCACATGG 3′, with an expected product of 775 nt.; and *Actin5C* (F) 5′-CTCACCTATAGAA**GACGAAGAAGTTGCTGCTCT**-3′ and (R) 5′ CTAACTGTTGAATC**CTCGTAGGACTTCTCCAACG**-3′, with an expected product of 747 nt. *Note:* for the *Actin5C* primers, only the nucleotides in **bold** match the cDNA sequence of *Actin5C*. Between 3 and 5 independent experiments were performed for each gene, with all PCR reactions run in triplicate. Graphed data represent average RT-PCR products of gene-specific primers relative to that of *Actin5C*, which was measured separately for every RT reaction. The intensities of the PCR bands were measured with ImageJ software; the data were prepared as graphs using GraphPad Prism 9.0.1.

### 4.4. Antibodies and Immuno-Fluorescence

Rabbit anti-*Drosophila* DCP1 [44] was provided by James Wilhelm and used at 1/2000 dilution. Guinea pig anti-*Drosophila* coilin, GP3 [53] was kindly provided by Dr. Joe Gall and used at 1/3000. Rat monoclonal anti-*Drosophila* Deadpan was purchased from abcam (#195173) and used at 1/150. Rabbit anti-Hid was kindly provided by Hyung Don Ryoo and used at 1/1000. We used two different antibodies against JNK; the antibody used in Figure 3A was a rabbit monoclonal antibody directed against phospho-JNK (Thr183/Tyr185) from Cell Signaling (#4668), while the antibody used in Figure S2 was a rabbit polyclonal anti-phospho-JNK was from Promega (#V7931). Rat monoclonal anti-*Drosophila* Hsc70.3 (BiP) was purchased from Babraham Bioscience Technologies, Cambridge, UK (clone MAC 143, #BT-GB-143P). Mouse monoclonal anti-β-tubulin (clone E7) was from the Developmental Studies Hybridoma Bank. Secondary antibodies included Alexa Flour 546 goat anti-guinea pig at 1/400 (A-11074, Thermo Fisher Scientific) and Alexa Fluor 647 donkey anti-rat at 1/300 (A-48272, Thermo Fisher Scientific). Ant-rabbit IgG-HRP and anti-mouse IgG-HRP were from Cell Signaling (#7074 and #7076, respectively). 

Tissues were dissected directly into a 2% formaldehyde solution [62]. All tissues were counter-stained with 4′, 6-diamino-2-phenylindole (DAPI, Polysciences) at 1 μg/mL prior to viewing. We used a Zeiss Axioskop equipped with a SPOT Pursuit digital camera and software, a Leica SP8 confocal microscope equipped with a white light laser, or an Olympus IXplore SpinSR spinning disk confocal microscope, the latter two instruments are in the Shared Instrumentation Facility at Louisiana State University.

### 4.5. EdU Labeling

Larval brains were dissected directly into phosphate-buffered saline, and within 5 min pulsed with 5-ethynyl-2-deoxyuridine (EdU) at 20 μM for 30 min at room temperature. Tissues were washed and fixed in 2% paraformaldehyde as described in the preceding section. A Click-iT kit employing Alexa Fluor 488 (C-10337, Invitrogen) was used according to the manufacturer’s instructions to detect the DNA-incorporated EdU.

### 4.6. Transmission Electron Microscopy

Routine fixation, LR White embedding, and thin sectioning of wild type and *KO121 Nopp140-/-* tissues were performed as described by He et al. [31]. We used a JEOL JEM-1400 TEM in the Shared Instrumentation Facility at Louisiana State University operating at 120 KV and its side-mounted Orius camera by Gatan. All images were batch-converted to TIFF files and prepared for publication using Adobe Photoshop.

## Supplementary Materials

The following are available online at https://www.mdpi.com/article/10.3390/ijms22136759/s1.
