# Supplementary material for: Drosophila to Explore Nucleolar Stress
Source: Int J Mol Sci. 2021 Jun 23;22(13):6759. doi: 10.3390/ijms22136759 (PMC8267670; doi:10.3390/ijms22136759)
Supplement: Supplementary file 1 [file ijms-22-06759-s001.zip › ijms-1256142-supplementary.pdf]

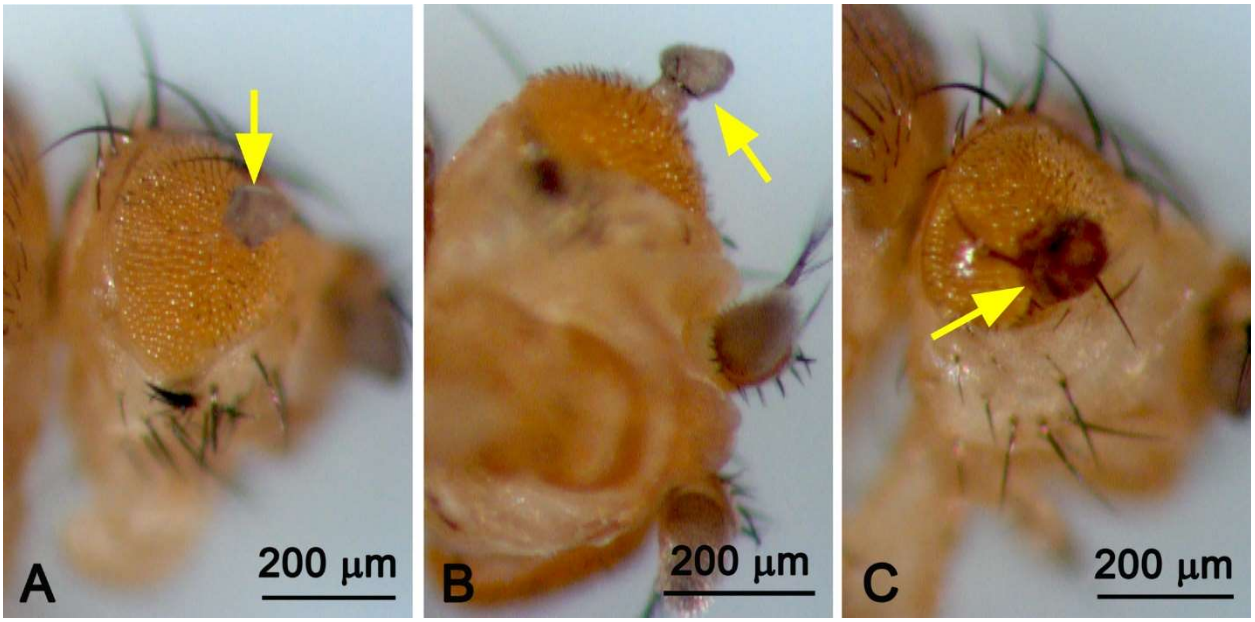

Figure S1. RNAi expression (*ey-GAL4>UAS-C4 RNAi*) depleted Nopp140 in larval eye-antennal discs resulting in unusual ectopic growths (yellow arrows) in the adult eyes. **(A, B)** The same fly tipped to get a better view of the ectopic tissue. **(C)** a separate fly with pigmented ectopic tissue. The precise nature of these ectopic tissues and their formation remain unknown.

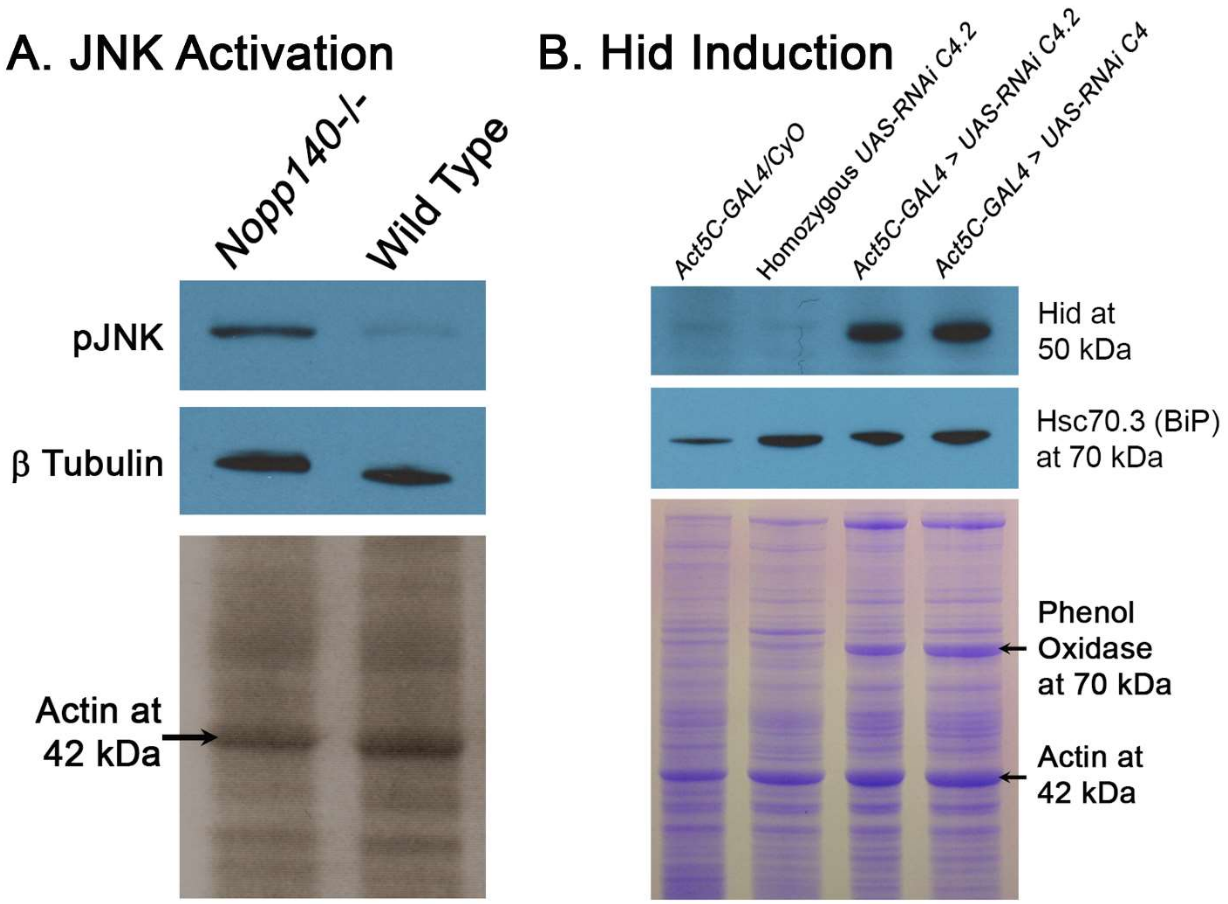

Figure S2. JNK pathway activation in Nopp140 knockout and knockdown larvae. **(A)** JNK upregulation was detected by anti-phospho-JNK (Promega) in *KO121 Nopp140-/-* larvae. Loading controls in companion gels included β-Tubulin (Western blot) and Actin (Coomassie). **(B)** Hid upregulation was detected in Nopp140-RNAi expressing larvae. Loading controls in companion gels included Hsc70.3 (BiP) (Western blot) and Actin (Coomassie). The Coomassie stained gel shows accumulations of phenol oxidase A3 at 70 kDa in larvae depleted for Nopp140. We originally identified the protein by mass spectroscopy [30], and its appearance correlates with the formation of melanotic 'tumors' in these larvae shortly before they die [60]. Phenol oxidases are released from hemolymph crystal cells [61] in a JNK-dependent manner [62].

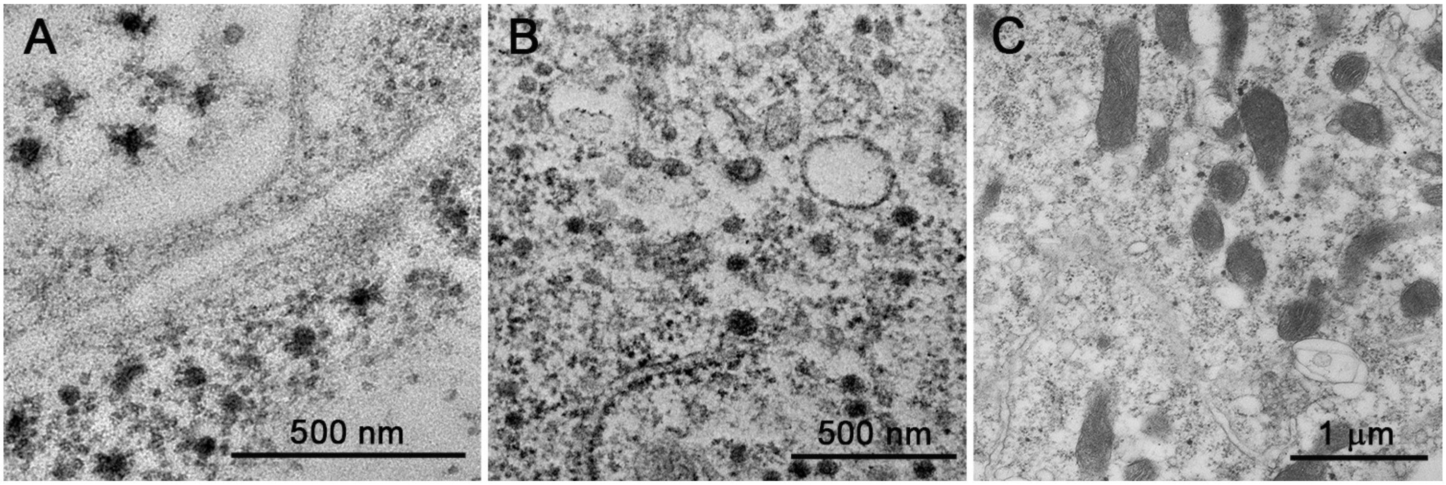

Figure S3. Transmission electron microscopy of dense granules found *J11 Nopp140-/-* larvae and in *Act5C > RNAi-C4* larvae. **(A)** Neuronal cells of the ventral nerve cord in *J11 Nopp140-/-* larvae. **(B)** Neuronal cells of the central brain lobe in *J11 Nopp140-/-* larvae. **C)** Midgut cells of *Act5C > RNAi-C4* larvae. In *J11 Nopp140-/-* larvae, the occurrence of neuronal cells containing the dense granules is low compared to the midgut in which every polyploid cell contains granules. The number of granules in midgut cells depleted for Nopp140 by RNAi was also reduced compared to midgut cell completely lacking Nopp140 by gene deletion or disruption (compare Figure 3 A, C).

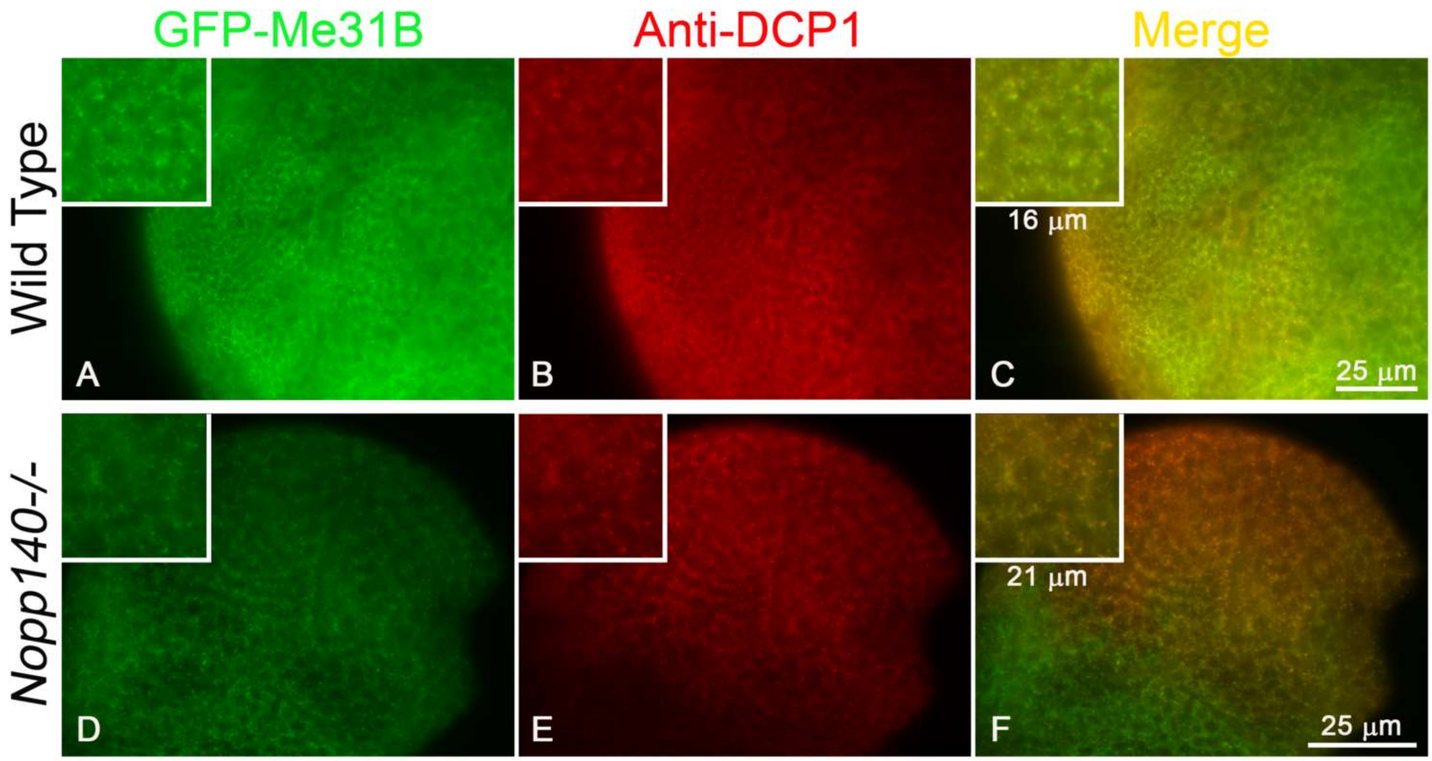

Figure S4. Colocalization of Processing (P) body markers in wild type neuronal mRNP granules. Both **(A-C)** Wild type (*w*[1118]) and **(D-F)** *KO121 Nopp140-/-* mRNP granules were concurrently labeled by P body markers GFP-Me31B and anti-DCP1 in the larval central brain lobe. (A-F) Bar = 25 µm. The insets provide high magnification views of (A-C) 16 µm across and (B-F) 21 µm across.
